# Leadership curricula and assessment in Australian and New Zealand medical schools

**DOI:** 10.1186/s12909-020-02456-z

**Published:** 2021-01-07

**Authors:** Simone Jacquelyn Ross, Tarun Sen Gupta, Peter Johnson

**Affiliations:** College of Medicine and Dentistry, James Cook University, Douglas, Queensland Australia

**Keywords:** Leadership, Medical leadership, Medical education, Medical student, Health system, Australia, Evaluation

## Abstract

**Background:**

The Australian Medical Council, which accredits Australian medical schools, recommends medical leadership graduate outcomes be taught, assessed and accredited. In Australia and New Zealand (Australasia) there is a significant research gap and no national consensus on how to educate, assess, and evaluate leadership skills in medical professional entry degree/programs. This study aims to investigate the current curricula, assessment and evaluation of medical leadership in Australasian medical degrees, with particular focus on the roles and responsibilities of medical leadership teachers, frameworks used and competencies taught, methods of delivery, and barriers to teaching leadership.

**Methods:**

A self-administered cross-sectional survey was distributed to senior academics and/or heads or Deans of Australasian medical schools. Data for closed questions and ordinal data of each Likert scale response were described via frequency analysis. Content analysis was undertaken on free text responses and coded manually.

**Results:**

Sixteen of the 22 eligible (73%) medical degrees completed the full survey and 100% of those indicate that leadership is taught in their degree. In most degrees (11, 69%) leadership is taught as a common theme integrated throughout the curricula across several subjects. There is a variety of leadership competencies taught, with strengths being communication (100%), evidence based practice (100%), critical reflective practice (94%), self-management (81%), ethical decision making (81%), critical thinking and decision making (81%). Major gaps in teaching were financial management (20%), strategic planning (31%) and workforce planning (31%). The teaching methods used to deliver medical leadership within the curricula are diverse, with many degrees providing opportunities for leadership teaching for students outside the curricula. Most degrees (10, 59%) assess the leadership education, with one-third (6, 35%) evaluating it.

**Conclusions:**

Medical leadership competencies are taught in most degrees, but key leadership competencies are not being taught and there appears to be no continuous quality improvement process for leadership education. There is much more we can do as medical educators, academics and leaders to shape professional development of academics to teach medical leadership, and to agree on required leadership skills set for our students so they can proactively shape the future of the health care system.

## Background

Health system reform models since the early 1990s have recommended leadership training for both medical students and doctors [1–7]. A clinical leader is expected to be able to recognise when health service change is required, motivate and inspire others to also do so, ensure the safety of the team’s action and outcomes, increase the ethical underpinnings of a health organisation and improve the quality of patient care [8–11].

In Australia, Health Workforce Australia, then the national health workforce agency, published a major report in 2013, the *Health LEADS Australia framework* in response to the perceived gap in medical leadership education and practice. This seminal report was the first national report and first national framework on medical leadership. While the Australian framework was written for health professionals in practice and not for medical professional entry degree/programs, it can, however, be utilised as key leadership training needs for medical education and across the Australian health system. The framework has a clear outline for working with others, and for promoting health system change. The framework outlines essential requirements for medical leadership training, including the five LEADS domains of *Leads Self, Engage Others, Achieve Outcomes, Drive Innovation, and Shape Systems* [12].

The Australian Medical Council (AMC), the accrediting body for medical schools, updated the *Standards for Assessment and Accreditation for Primary Medical Programs* in 2012 [13] and included a new domain of professionalism and leadership. Described within this domain, are ten graduate outcomes with two relating to demonstrating or describing the qualities and principles of leadership (4.2 and 4.3). Another two refer to desirable qualities of medical leadership, such as being an effective inter-professional team member (4.8) and educating colleagues for patient care (4.9). While the AMC allows medical professional entry degrees/programs flexibility in how they meet these graduate outcomes, this evidence suggests the AMC expects leadership education is to be taught, assessed and evaluated in all medical schools.

There are significant gaps in the research literature on how to educate, assess, and evaluate leadership skills in medical professional entry degree/programs. Numerous authors in the United Kingdom [14], United States [15] and Australia [16] have described the paucity of research in the teaching and assessment of leadership skills training across the continuum of medical education. Teaching of leadership in medical degrees is often not compulsory, with the curricula developed without assessable leadership competencies [14–17]. From an education perspective, McKimm [18] recently noted that ‘*leadership practice and development … needs to be evidenced based, theory informed and practice driven’*. The current authors have recently argued [19] that leadership in practice can only occur for students if the organisational structure and culture of medical schools, hospital services and private practices allows students the opportunity to learn to lead and to practise. From an assessment and evaluation perspective, Lees and Armit [20] recently asserted that ‘*medical leadership enjoys less respect within the industry, [with] minimal research funding*’. This can have a detrimental impact on the development of any new curricula, let alone one that is required to be taught, assessed and evaluated.

As there is no Australian and New Zealand (Australasian) national consensus on when or how to teach, assess, or evaluate leadership in a medicine curriculum, it is clear that there is much we can do as medical educators, academics and leaders to shape a medical leadership curricula and agree on the required skills taught and assessed in Australasian medical professional entry degree/programs. This paper describes the results of a medical leadership curricula, assessment and evaluation survey of medical professional entry schools in Australasia. This survey sought to examine:

1. the roles and responsibilities of medical leadership teachers;
2. medical leadership frameworks and usage in the curricula;
3. medical leadership competencies taught under the Health LEADS Australia domains;
4. current methods of delivery and opportunities for student feedback;
5. methods of assessment and the competencies assessed;
6. barriers to leadership education, assessment, and evaluation;
7. the support required to integrate or assess leadership in the curriculum.

## Methods

In Australasia, there are a variety of medical professional entry degree/programs including undergraduate entry or graduate entry, or a mix of both. In this paper, the term *undergraduate entry* refers to a university tertiary degree where the entry requirement is to have completed and hold a secondary school qualification, whereas *graduate entry* refers to a university tertiary degree where the entry requirement is to have already completed and hold a tertiary university degree qualification. Also, in this paper, the terms medical school or programs/degrees, refers to a qualification that permits the holder to seek general registration as a medical practitioner and does not refer to specialist (postgraduate) training.

A self-administered cross-sectional survey was distributed to senior academics and/or Heads or Deans of Australasian medical schools. Data was collected over an eight-month period. In November 2018 a letter of invitation to complete an online survey was emailed to an email alias of senior academic medical school staff in Australasian medical schools. In July 2019, a second letter of invitation was then sent via email to medical Deans or Heads of schools who had not completed the survey. To ensure consistency of data, the same survey was administered.

### Survey development

Survey questions were based on a review of medical leadership articles, documents and surveys. The United Kingdom Faculty of Medical Leadership and Management (FMLM) Curricula Study – Interview Guide [14] was used as a foundation for the survey. According to Jefferies et al., the FMLM Curricula Study – Interview Guide (2017, p1095) was created to “*establish a picture of leadership and management in education in undergraduate medical curricula at all UK medical schools*”. The survey questions were adapted to be more specific to Australasian medical leadership requirements, and medical education, assessment and evaluation. Questions covered: background; current leadership curricula and development and potential barriers; student assessment, evaluation and potential barriers; and questions to inform national leadership competencies across the medical education continuum from selection to graduate education. Questions asking about the teaching of leadership were organised by the Health LEADS Australia Domains [12] (*1. Leads Self, 2. Engages with Others, 3. Achieves Outcomes, 4. Drives Innovation, and 5. Shapes Systems*). As the Health LEADS Australia framework does not provide competencies, each of these five domains were further categorised with leadership and management competencies as described in the book ‘Leading and Managing Health Services: An Australian Perspective’ [21]. (See Table 1) Academics were asked to categorise at what level they believed the leadership competencies (skills) were taught in their medical degree, from *Not At All, Introduced, Reinforced,* and *Mastered* to determine the achievement of a specified competency, or as Harden (2009, pg678) states “the level of mastery of a subject area” [22]. Ethics approval was obtained through the JCU Human Ethics Committee (H6985).

**Table 1:** Medical Leadership Domains and Competencies

Health LEADS Australia Domains	Leads Self	Engages with Others	Achieves Outcomes	Drives Innovation	Shapes Systems
Leading and Managing Health Services: An Australian Perspective Competencies	Ethical decision making	Communication	Critical thinking and decision making	Creativity and visioning	Workforce planning
	Self-management	Inter-professional teamwork	Managing staff	Evidence-based practice and use	Strategic planning
	Emotional intelligence and self-awareness	Partnering with stakeholders	Project management	Successfully managing conflict	Health service planning
	Exploring values	Power in organisations	Financial management	Building positive workplace culture	
	Critical reflective practice	Networking	Negotiation	Leadership and management of change	
				Quality and service improvement	

### Data analysis

Data were entered into Microsoft Excel 2016. Data for closed questions (yes, no) and the ordinal data of each Likert scale response (*Not At All* covered, *Introduced*, *Reinforced*, and *Mastered*) were described via frequency analysis, with the Likert scale responses summarised in pie and bar graphs. A deductive analysis of the self-reported leadership competencies taught, was conducted using the Health LEADS Australia domain headings as coding categories, as described in Fig. 6. Content analysis of the free text responses to barriers to teaching, was undertaken by author SR and confirmed by authors TSG and PJ, using an inductive, iterative process to identify codes and categorise them into overarching themes. The frequencies of the responses are described in the results section and overarching themes are described in Fig. 7 [23]. For bivariate analysis comparing leadership competencies of undergraduate versus graduate entry, the data was imported into the computerised Statistical Package for Social Sciences (SPSS) release 23 for Windows (http://www.spss.com) and assessed using Student’s t-tests. Frequencies are described in the results section.

## Results

Of the 23 medical degrees, three (13%) did not respond at all, one (4%) was removed at their own request as it is a new degree and they felt it was inappropriate to participate, two (9%) gave no further details after the degree demographics, and one (4%) gave no further details after the question which asked ‘do you teach leadership at your school’ in which they reported that medical leadership was not currently taught in their degree (6%). Full surveys were completed for sixteen (73%) medical degrees. Nine of the 16 degrees (56%) are graduate entry only, seven (44%) undergraduate, and three (19%) are mixed with the entry dependent on whether the student is a graduate entry or school leaver.

The following section outlines the results for the medical leadership curricula survey, including: roles and responsibilities; leadership education; assessment and evaluation; overall barriers; and reported needs to integrate or assess leadership curricula.

### Leadership curricula

#### Roles and responsibilities

Of the sixteen medical degrees teaching medical leadership, one (6%) degree has a standalone academic lead teaching leadership, eight (50%) have a lead that is combined with another role, seven (44%) do not have a lead. A variety of staff are responsible for delivering leadership training including: academic faculty (16, 100%); clinical faculty (11, 69%); hospital and health service staff – clinical (11, 69%); hospital and health service staff – educators (6, 38%); and external providers or third party leadership development consultants (4, 25%).

#### Leadership education

Leadership teaching is taught as a common thread integrated throughout the entire curricula or across several subjects (11, 69%), via ad-hoc teaching (7, 44%), a specific module for all students (2, 13%), or a specific module as an elective (2, 13%). There are plans to introduce or make change to the curricula to integrate leadership more generally at 12 (71%) degrees; 2 (12%) are doing so within the next 6 months, 4 (24%) between six to twelve months, and 6 (35%) between one to 2 years. Students will have input into these changes via student-staff committees (14, 82%), medical student association input (13, 76%), student satisfactions surveys (10, 59%), and student focus groups (3, 18%).

Australian and overseas resources used to inform the leadership curricula of the degrees include:

- AMC Professionalism and Leadership Graduate Domain and Statement (Australia) 2012 [13] – 14 (88%)
- Good Medical Practice: A Code of Conduct for Doctors in Australia 2014 [24] – 13 (81%)
- Health Leads Australia Framework 2013 [12] – 5 (31%)
- Medical Leadership Competency Framework: Enhancing Engagement in Medical Leadership (NHS, UK) 2010 [25] – 2 (13%)
- CanMEDS: Better Standards, Better Physicians, Better Care (Canada) 2015 [26] – 2 (13%)
- Australian Commission on Safety and Quality in Health Care [27] – 1 (6%)
- Leading and Managing Health Services: An Australian Perspective 2015 [21] – 1 (6%)
- General Medical Council: Leadership and Management for Doctors (UK) 2012 [28] – 1 (6%)
- Kings Fund: Leadership and Leadership Development in Health Care (UK) 2015 [29] – 1 (6%)

**Fig. 1:**
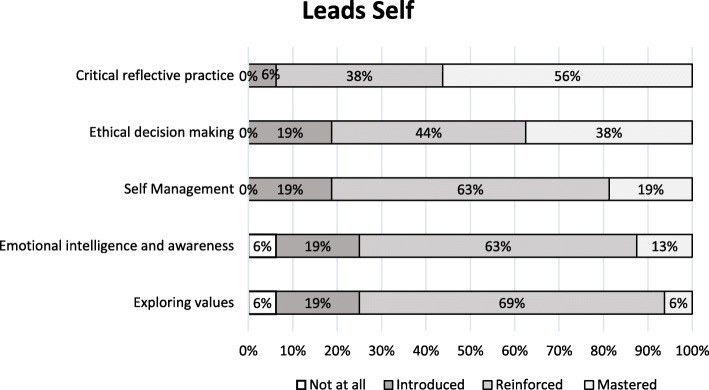
Leading Self: Competencies taught and level of teaching in Australasian medical courses

**Fig. 2:**
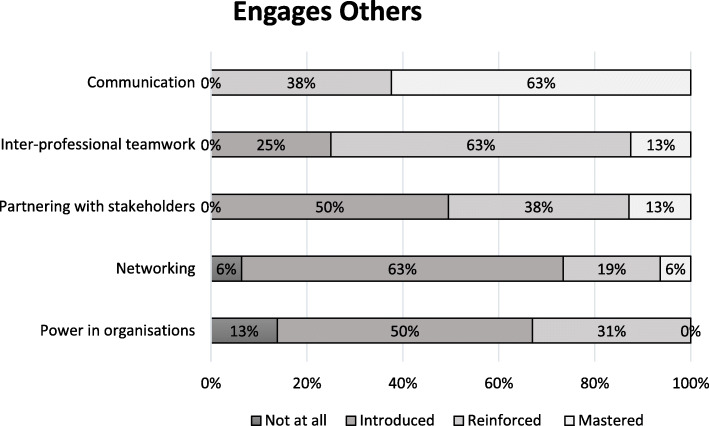
Engages Others: Competencies taught and level of teaching in Australasian medical courses

**Fig. 3:**
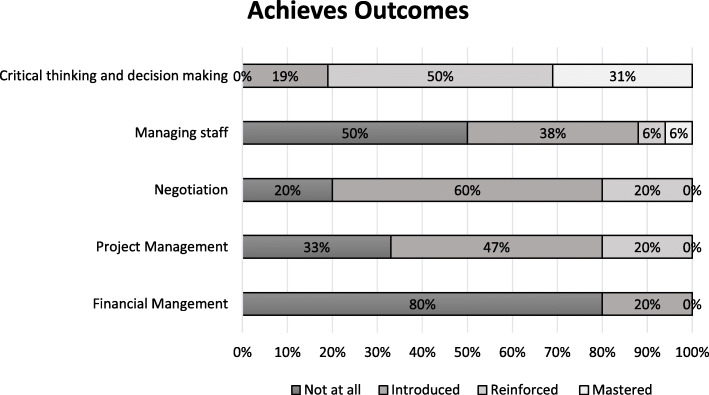
Achieves Outcomes: Competencies taught and level of teaching in Australasian medical courses

**Fig. 4:**
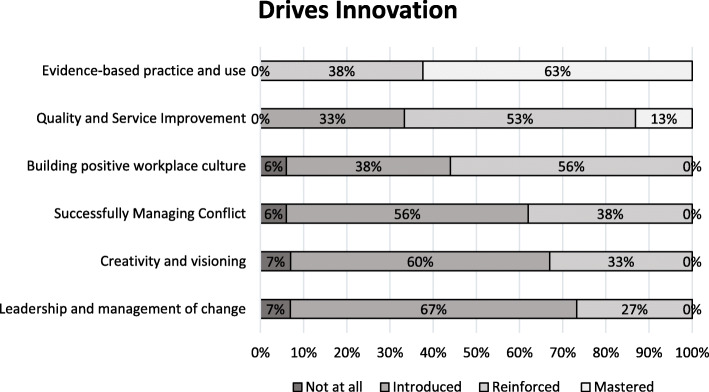
Drives Innovation: Competencies taught and level of teaching in Australasian medical courses

**Fig. 5:**
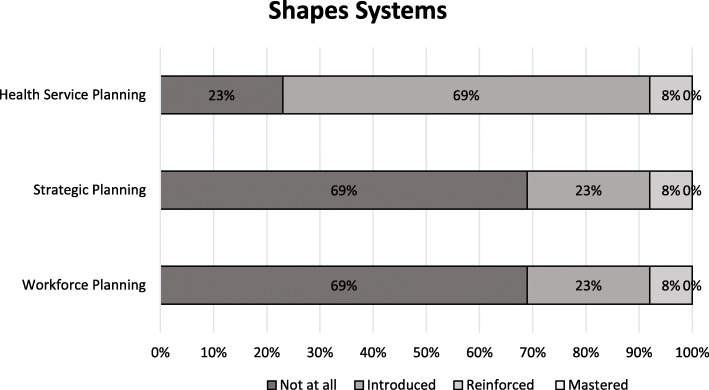
Shapes Systems: Competencies taught and level of teaching in Australasian medical courses

Figures 1, 2, 3, 4 and 5 show a self-reported snapshot of what competencies are taught and at what level in Australasian medical courses. These figures show the variation of teaching within medical degrees with some clear strengths and gaps. For example, competencies that are taught at *Reinforced* or *Mastered* level for more than 50% of the degrees are: communication (100%); evidence-based practice and use (100%); critical reflective practice (94%); self-management (81%); ethical decision making (81%); critical thinking and decision making (81%); exploring values (75%); emotional intelligence and awareness (75%); inter-professional teamwork (75%); quality and service improvement (67%); building positive workplace culture (56%); and partnering with stakeholders (50%). Competencies covered by less than 50% of the degrees are financial management (20%), strategic planning (31%), and workforce planning (31%).

**Fig. 6:**
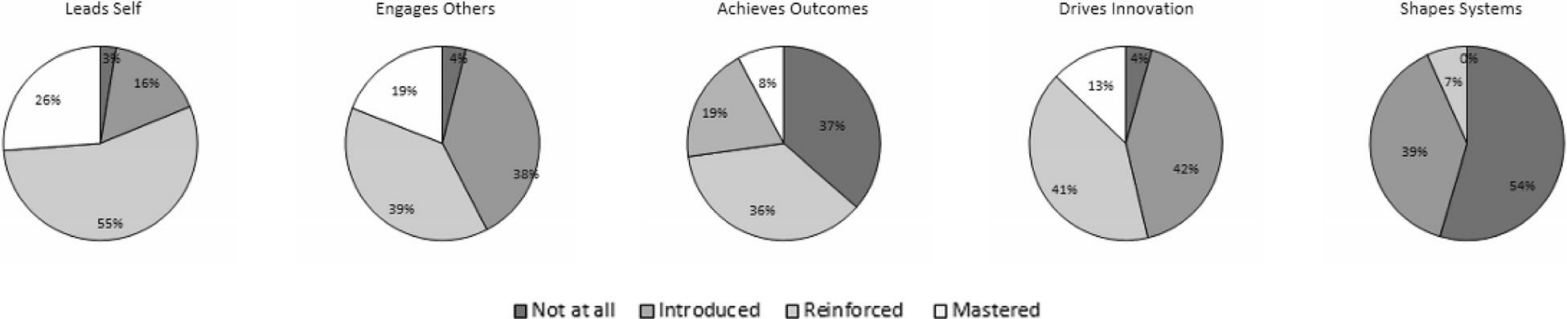
Domains and level of teaching in Australasian medical courses

Fourteen (88%) medical degrees taught all competencies in *Lead Self*, 13 (81%) in *Engages Others*, two (13%) in *Achieves Outcomes*, and 13 (815%) in *Drives Innovation*. Of the 13 degree responses for *Shapes Systems*, two (15%) taught every leadership competency. One medical degree reported teaching every leadership competency across all domains of *Leads Self, Engages Others, Achieves Outcomes, Drives Innovation*, and *Shapes Systems*. Leadership teaching also was reported as not being taught in one medical degree. A comparison of leadership competencies for undergraduate entry versus graduate entry degrees did not reveal any statistical significance differences. However, no undergraduate entry degrees taught health workforce planning compared to four (44%) graduate entry degrees. Figure 6 shows an overview of what is taught and at what level in Australasian medical professional entry degree/programs.

The teaching methods used to deliver medical leadership education are diverse. So too are other opportunities outside the curricula for students to learn medical leadership. (See Table 2).

**Table 2:** Medical Leadership Teaching and Assessment Methods used in Australasian medical courses

Medical leadership teaching methods in the curricula (*N* = 16)	Other opportunities outside the curricula for students to learn medical leadership (*N =* 16)	Formal assessment of medical leadership in the curricula (*N* = 10)
Small group seminars or workshops (14, 88%)	Student clubs and societies (14, 82%)	Reflective writing (10, 100%)
Experiential learning (14, 88%)	Peer-teaching (4, 24%)	Portfolio (6, 60%)
Lectures (11, 69%)	Scholarships (3, 18%)	Mini-CEX (6, 60%)
Problem-based learning (7, 44%)	Sitting on school committees (2, 12%)	Structured clinical assessments (e.g. OSCE) (5, 50%)
Student selected components (3, 19%)	Attending leadership conferences (2, 12%)	Presentations (5, 50%)
Opportunity for a selective subject (2, 12%)	Coursework overload study (2, 12%)	Written examinations (5, 50%)
Group project (1, 6%)	Mentoring (1, 6%)	Case-based discussions (4, 40%)
Case-based learning (1, 6%)		
Student suggestions for a guest speaker (1, 6%)		
Attending simulation sessions (1, 6%)		

#### Assessment and evaluation of leadership education

Seven (41%) of the seventeen responses formally evaluate medical leadership teaching at their school. Barriers to evaluating student leadership competencies are described in the next section.

Ten (59%) of the seventeen responses formally assess medical leadership. (See Table 3). Four (44%) degrees also provide a faculty generated score of professional behaviour for students. Of the ten responses received, there was a variety of competencies assessed by multiple degrees including communication (6, 67%), quality and safety (3, 38%), and ethical practice (3, 33%).

A deductive content analysis of the competencies using the Health LEADS Australia domain headings [12] is found in Table 3. The below competencies assessed by domain are similar to the competencies taught in Fig. 6, with *Leads Self*, Engage *Others*, and *Drives Innovation* being taught and assessed more than *Achieves Outcomes* and *Shapes Systems*.

**Table 3:** Domains and competencies assessed in Australasian medical courses

Leads Self n (%)	Engage Others n (%)	Achieves Outcomes n (%)	Drives Innovation n (%)	Shapes Systems n (%)
Ethical practice (3, 33%)	Communication (6, 67%)	Critical thinking and decision making (1, 11%)	Quality and safety (3, 33%)	
Self-management (1, 11%)	Teamwork (1, 11%)		Evidence based practice (1, 11%)	
Self-awareness (1, 11%)			Conflict Management (1, 11%)	

#### Barriers to leadership education, assessment and evaluation

All sixteen responses (100%) reported at least one barrier to integrating leadership material in the curriculum. The overarching themes shows the barriers varied, with the most common barrier being competition for teaching time in the curriculum (8, 47%). Other responses provided by more than one degree included a lack of national curricula or guidelines (6, 35%), timetabling (3, 18%), a lack of expertise in teaching leadership (2, 12%) and/or a perspective the students are not yet mature enough to comprehend the significance of leadership (2, 12%).

**Fig. 7:**
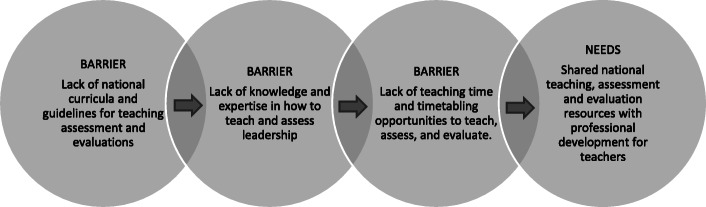
Key barriers and needs for leadership education, assessment and evaluation

All sixteen responses (100%) reported one or more barriers to assessing leadership in the curriculum. Responses provided by more than one degree to assessment barriers included: lack of formal teaching in the curricula (5,29%); lack of knowledge about how to teach and assess leadership (4, 24%); assumption that students already have leadership knowledge and do not need it taught or assessed (3, 18%); too many other assessments (2, 12%); the subjective nature of the content (2, 12%); lack of suitable assessment instruments (2, 12%); and lack of identified curriculum (2, 12%). See Fig. 7.

All sixteen (100%) responses reported one or more barriers to evaluating leadership competencies, with one (6%) response stating ‘unsure’. Responses provided by more than one degree to evaluation barriers, included: lack of suitable evaluation instruments (3, 19%); time (3, 19%); pressure to evaluate other curricula components (3, 19%); not sure what competencies should be evaluated (2, 13%); and/or lack of leadership learning outcomes (2, 13%).

#### School-reported needs to integrate or assess leadership in the curricula

There were sixteen responses to the question asking what support would be helpful for medical degrees to integrate or assess leadership in the curriculum (See Fig. 7). The overarching themes included: shared national teaching, assessment and evaluation resources (11, 69%); agreed national curricula with learning outcomes (8,50%); more funding for staff to develop leadership programs (3, 19%); professional development for teachers on content and teaching (2, 13%); and renaming leadership education to leadership and management (1, 6%).

## Discussion

This is the first in-depth study into medical leadership teaching, assessment and evaluation practice in medical degrees in Australia and New Zealand. As the full survey was completed for 16 (73%) medical degrees these results give a fairly complete picture of leadership curricula. The findings are relevant for the AMC, agencies that teach leadership skills in clinical contexts, medical educators, clinical educators, academics, students, and the public.

Of the 17 degrees that completed survey questions beyond the demographic questions, one (6%) responded they do not teach medical leadership. Sixteen (94%) responded they teach leadership skills training, with formal leadership training occurring for more than three-quarters of these degrees. There is a wide diversity of staff delivering the education, such as academics, clinical staff and external providers or third party leadership development consultants, with students heavily involved in providing feedback for curricula change. Two-thirds of the schools have future plans to introduce or make changes to the curricula to integrate this topic more generally. However, overall, the assessment and evaluation of the medical leadership curricula is ad-hoc with only half assessing the leadership curricular content and only one-third evaluating it. This indicates for two-thirds of the Australasian degrees there is no continuous quality improvement occurring for leadership education.

With the recent leadership education findings from the UK [14], Australasian medical leadership education is on-par with the UK for teaching leadership (94% compared to 92%), evaluating the skills (42% compared to 48%), but is lower for assessment (59% compared to 75%) of these skills. Recent US findings [15], suggested leadership was taught far less commonly than in Australasian medical schools (54% compared to 94%) with no US data available to compare leadership skills evaluation or assessment. The systematic review by Webb et al., of leadership teaching in undergraduate medical education [17] found that most leadership curricula did not demonstrate student behaviour change, as often the curricula is taught without the use of a leadership framework and without evaluating leadership competencies, and therefore is also lacking continuous quality assurance of the teaching and learning of students.

### Leadership competencies

The competencies in the survey were chosen to be relevant for medical leadership in the Australasian health service. For example, communication with patients require skills such as the ability to actively listen, problem solve, and be patient-centred. In contrast, leadership within the health service requires different communication skills including inter-professional communication of active listening, knowledge of the organisations communication protocols, when and how to use formal or informal communication, plus knowing how and when to have a difficult conversation with team members [30]. Lack of knowledge of these skills in the work environment can increase or create unhealthy working environments which impact on patient safety [9].

In most Australasian degrees, the current competencies taught at *Reinforced* or *Mastered* levels are providing a well-constructed student understanding of professionalism, which is the basis for a good leader and a good doctor. However, it appears that some of the key leadership competencies are rarely being taught. These include: leadership and management of change, successfully managing conflict, negotiation, project and financial management, and an understanding of power in an organisation. This means we are teaching students to lead themselves and engage with others, and to be an advocate, but we are not providing them with the knowledge and potentially the transformative leadership tools to fully drive innovation. An understanding of the underlying health needs and systems are vital if students are to drive a medical innovation. With appropriate leadership training, graduating students should be able to implement organisational based changes including building positive workplace culture, which creates a healthy working environment, and increases staff job satisfaction and the safety of patients [9, 31–33]. These are skills required for the rest of their career.

### Barriers and needs

The most common reported barrier to leadership education is teaching time in the curricula. Miller, Till and McKimm, 2018 [34], state that “*medical students, like all health-care professionals, can ‘learn to lead’ and they should be supported to do so, despite an already crowded undergraduate medical degree”.* Based on further reported barriers, such as a lack of national curricula and a lack of expertise or knowledge to teach leadership, the authors recommend that academics should also be supported, nationally and locally, to develop resources and align content. There is a strongly reported need for shared national medical leadership teaching, assessment and evaluation resources with professional development for teachers. To assist with this change, the authors recommend a health leadership core curriculum and teaching methods with a leadership development real-world work integrated focus. This would align with other health teaching domains, such as professionalism [24], ethics [35], Indigenous health [36], as well as most health science disciplines [13]. The development of a core leadership curriculum and teaching methods requires a ‘meeting of the minds’ to discuss key competencies for medical leadership teaching, for both the professional development of academics and for the curricula knowledge and skills learning for students.

### Limitations

The UK tool used as a foundation for the survey had been designed for the UK undergraduate medical school context. While this has not been validated in the Australasian context, the survey questions were adapted to be more specific to Australasian medical education and the findings report similar results. The survey was administered twice over an eight-month period with one question re-sent to original survey recipients. This process occurred to ensure the appropriate member of faculty completed the data collection. Due to the high response rate (73%) with 16 of 22 eligible medical schools completing the full survey, the data was unlikely to be significantly affected by selection bias even though it was self-reported and survey responses were not triangulated with other curriculum documents or maps. This data was collected from primary medical programs and does not include data from students or clinical leadership training organisations, however data is currently being collected from these population groups.

## Conclusion

In the majority of Australasian medical degrees, medical leadership is being taught, but there is variability in the medical leadership training, evaluation and assessment in each degree. Competencies being taught at *Mastered* provide a good basis of professionalism in most medical degrees, but key leadership competencies of change management, conflict management, negotiation, managing staff and understanding power in an organisation are not. A meeting of minds to discuss a national medical leadership curricula is recommended.

This study builds on the limited knowledge regarding basic medical leadership education in Australasia. Added to the recent medical leadership curricula data from the UK [14] and US [15] and based on the health system reform models since the 1990s recommending leadership training for medical students [1–7], it is clear nationally and globally that requirements include evidence based guidelines for teaching medical leadership, with curriculum that is clear and transparent, linked to industry and future practice, and appropriately assessed and evaluated. Further, local recommendations should include professional development for teaching medical leadership. This provides the opportunity to build effective academic leadership in our teachers and provide students with the skills and knowledge for leadership roles in the future.

## Supplementary Information

**Additional file 1.**

## Data Availability

All data generated or analysed during this study are included in this published article, and its supplementary information files.
