# Supplementary material for: Leadership curricula and assessment in Australian and New Zealand medical schools
Source: BMC Med Educ. 2021 Jan 7;21:28. doi: 10.1186/s12909-020-02456-z (PMC7792303; doi:10.1186/s12909-020-02456-z)
Supplement: Supplementary file 1 — Additional file 1. [file 12909_2020_2456_MOESM1_ESM.pdf]

Medical Leadership Curricula, Assessment and Evaluation Survey

Welcome to the Australian Medical Schools Leadership Curricula, Assessment and Evaluation Survey

RESEARCH PROJECT
You are invited to take part in a research project to establish an overview of medical leadership education in basic medical education curricula at all Australian schools, to assess the current content and methods of leadership education and assessment, and to understand which resources are being used to support the development of the curricula.

RESEARCHERS INVOLVED
Ms Simone Ross is conducting this survey under the supervision of Prof Tarun Sen Gupta and A/Prof Peter Johnson at James Cook University as part of a PhD in medical education. Human Ethics Committee (H6985) at James Cook University has approved the study.

CONFIDENTIALITY
This survey is confidential and results will be anonymized, however, there is opportunity for you to share best practice and demonstrate the strength of your curriculum.

QUESTIONNAIRE
This survey covers background, current leadership curricula and development, student assessment, evaluation and potential barriers, as well as questions to inform national leadership competencies across the medical education continuum from selection to graduate education. The results from this study will be used to guide the development of a national ideal curricula content, format, delivery method, duration and/or timing of leadership education.

This survey has been adapted for Australia from the Faculty of Medical Leadership and Management Curricula Study in the United Kingdom.

PREFER TO BE INTERVIEWED?
If you would prefer not to complete this survey and instead have a face-to-face interview via video-conference, please email me at simone.ross@jcu.edu.au and let me know some times and dates that suit you.

CONSENT TO COMPLETE SURVEY
When you click on the NEXT button below, you consent to participate in this project. You also acknowledge that taking part in this study is voluntary, you can stop at anytime without explanation or prejudice, and all information will be kept strictly confidential/anonymous. The data from the study will be used in research publications and reports. You and your school will not be identified in any way in these publications and reports.

Medical Leadership Curricula, Assessment and Evaluation Survey

General Questions

This section has general questions about the medical school and the degree program

* 1. Name of medical school

* 2. Your full name

3. Your job title

* 4. Name of degree program

5. How many years of medical school are taught at this university?

- 3
- 4
- 5
- 6
- Other (please specify)

* 6. Is it undergraduate and/or postgraduate entry?

- Undergraduate only
- Postgraduate only
- Mixed Undergraduate/Postgraduate (please specify)

Medical Leadership Curricula, Assessment and Evaluation Survey

Leadership Curricula and Development Barriers

The following questions assess the current leadership curriculum development barriers

* 7. Is medical leadership taught in your medical degree?

- Yes
- No

* 8. What have been / might be the potential barriers to integrating leadership material into your curriculum?

Medical Leadership Curricula, Assessment and Evaluation Survey

Leadership Curricula and Development

The following questions assess the current leadership curricula development process. Thus, we are asking questions about how the curriculum was developed, the leadership skills covered, and how it is taught in the program.

* 9. Are any of the following resources used to inform your leadership curricula? Select all that apply

- [ ] AMC Professionalism and Leadership Graduate Domain and Statement
- [ ] Health Leads Australia Framework
- [ ] Leading and Managing Health Services: An Australian Perspective (book)
- [ ] Good Medical Practice: A Code of Conduct for Doctors in Australia
- [ ] Other? (please describe)

* 10. Leads Self: To what level do you feel each of the below leadership skills are covered in your medical school curriculum? (1. Day GE, Leggat SG. Leading and Managing Health Services: An Australian Perspective. 2015, Cambridge University Press. 2. Health LEADS Australia: The Australian Health Leadership Framework)

	Not at all covered	Introduced	Reinforced	Mastered	Couldn't say
Ethical decision-making	○	○	○	○	○
Self-management	○	○	○	○	○
Emotional intelligence and self-awareness	○	○	○	○	○
Exploring values	○	○	○	○	○
Critical reflective practice	○	○	○	○	○

* 11. Engages Others: To what level do you feel each of the below leadership skills are covered in your medical school curriculum? (1. Day GE, Leggat SG. Leading and Managing Health Services: An Australian Perspective. 2015, Cambridge University Press. 2. Health LEADS Australia: The Australian Health Leadership Framework)

	Not at all covered	Introduced	Reinforced	Mastered	Couldn’t say
Communication	◯	◯	◯	◯	◯
Inter-professional teamwork	◯	◯	◯	◯	◯
Partnering with stakeholders	◯	◯	◯	◯	◯
Power in organisations	◯	◯	◯	◯	◯
Networking	◯	◯	◯	◯	◯

* 12. Achieves Outcomes: To what level do you feel each of the below leadership skills are covered in your medical school curriculum? (1. Day GE, Leggat SG. Leading and Managing Health Services: An Australian Perspective. 2015, Cambridge University Press. 2. Health LEADS Australia: The Australian Health Leadership Framework)

	Not at all covered	Introduced	Reinforced	Mastered	Couldn’t say
Critical thinking and decision-making	◯	◯	◯	◯	◯
Managing staff	◯	◯	◯	◯	◯
Project management	◯	◯	◯	◯	◯
Financial management	◯	◯	◯	◯	◯
Negotiation	◯	◯	◯	◯	◯

* 13. Drives Innovation: To what level do you feel each of the below leadership skills are covered in your medical school curriculum? (1. Day GE, Leggat SG. Leading and Managing Health Services: An Australian Perspective. 2015, Cambridge University Press. 2. Health LEADS Australia: The Australian Health Leadership Framework)

	Not at all covered	Introduced	Reinforced	Mastered	Couldn’t say
Creativity and visioning	◯	◯	◯	◯	◯
Evidence-based practice and use	◯	◯	◯	◯	◯
Successfully managing conflict	◯	◯	◯	◯	◯
Building positive workplace culture	◯	◯	◯	◯	◯
Leadership and management of change	◯	◯	◯	◯	◯
Quality and service improvement	◯	◯	◯	◯	◯

* 14. Shapes Systems: To what level do you feel each of the below leadership skills are covered in your school curriculum? (1. Day GE, Leggat SG. Leading and Managing Health Services: An Australian Perspective. 2015, Cambridge University Press. 2. Health LEADS Australia: The Australian Health Leadership Framework)

	Not at all covered	Introduced	Reinforced	Mastered	Couldn't say
Workforce planning	☐	☐	☐	☐	☐
Strategic planning	☐	☐	☐	☐	☐
Health service planning	☐	☐	☐	☐	☐

15. Are there any other leadership skills covered in your medical school? If yes, to what extent do you feel they are covered?

* 16. To what level do you feel the five domains of the Health LEADS Australia Framework are covered in your medical school curriculum?

	Not at all covered	Introduced	Reinforced	Mastered	Couldn’t say
Leads Self	◯	◯	◯	◯	◯
Engages Others	◯	◯	◯	◯	◯
Achieves Outcomes	◯	◯	◯	◯	◯
Drives Innovation	◯	◯	◯	◯	◯
Shapes Systems	◯	◯	◯	◯	◯

* 17. To what level do you feel the AMC leadership graduate outcomes are achieved in your medical school curriculum?

	Not at all	Introduced	Reinforced	Mastered	Couldn't say
4.2 Demonstrate the qualities of integrity, honesty, leadership and partnership to patients, the profession and society	◯	◯	◯	◯	◯
4.3 Describe the principles and practice of professionalism and leadership in health care	◯	◯	◯	◯	◯
4.8 Work effectively as member of an inter-professional team	◯	◯	◯	◯	◯
4.9 Learn fundamental skills in educating colleagues for patient care	◯	◯	◯	◯	◯

* 18. Does the medical school have an academic lead for leadership in the curricula?

- ◯ Yes - It is a stand alone role
- ◯ Yes - It is combined with another role
- ◯ No
- ◯ Other (please specify)

* 19. Who is responsible for delivering training on leadership? Select all that apply

- ☐ Academic faculty
- ☐ Clinical faculty
- ☐ Hospital and health services staff - clinical
- ☐ Hospital and health services staff - educators
- ☐ External providers

Please elaborate

* 20. How is the leadership taught?

- ☐ As a common thread carried throughout the entire curricula or across several subjects
- ☐ Specific module undertaken by all students
- ☐ Specific module undertaken as elective
- ☐ Ad hoc

Please elaborate

* 21. What teaching methods does your medical school use to deliver leadership education? Select all that apply

- ☐ Problem based learning
- ☐ Lectures
- ☐ Small group seminars or workshops
- ☐ Experiential learning
- ☐ Student selected component
- ☐ Double degree with leadership
- ☐ Other (please specify)

Potential for other leadership education

The following questions are designed to determine opportunities for extra-curricula leadership learning, any potential for change to the leadership curriculum, and the input that students will have in any changes.

* 22. What other opportunities are there for students to learn leadership in your medical school (e.g. student clubs and societies)

* 23. Are there plans to introduce or make changes to your curriculum to integrate leadership more generally?

- Yes - within the next 6 months
- Yes - between 6 to 12 months
- Yes - between 1 to 2 years
- Other (please specify)
- Don't Know
- No

* 24. Please specify in what way do students have input into these changes? Select all that apply

- No student input
- Student focus group
- Medical student association input
- Student-Staff committee
- Student satisfaction survey
- Don’t know
- Other (please specify)

Medical Leadership Curricula, Assessment and Evaluation Survey

Leadership Barriers

The following questions are designed to assess potential barriers to assessing and evaluating leadership

* 25. Do you currently assess or evaluate medical leadership at your school?

- [ ] Yes - Assessment
- [ ] Yes - Evaluation
- [ ] No

* 26. What have been / might be the potential barriers to assessing leadership in your curriculum?

* 27. What have been / might be the potential barriers to evaluating students leadership competencies in your curriculum?

Leadership Assessment

The following questions are designed to assess student assessment of the current leadership curricula. Thus, we are asking questions about how leadership competencies are assessed.

* 28. How do you assess medical leadership competencies at your school? Select all that apply

- [ ] Portfolio
- [ ] Logbook
- [ ] Reflective writing
- [ ] Feedback – tutor
- [ ] Feedback – multisource
- [ ] Mini-CEX
- [ ] Professional behavior score
- [ ] Project report
- [ ] Audit (report)
- [ ] Case-based discussion
- [ ] Structured clinical assessments (e.g. OSCE)
- [ ] Meeting course requirements
- [ ] Presentation sessions
- [ ] Written examinations
- [ ] Not applicable
- [ ] Other (please specify)

29. Please describe the leadership competencies that these items assess

Medical Leadership Curricula, Assessment and Evaluation Survey

National Leadership Competencies

The following questions are designed to inform national leadership competencies across the medical education continuum from selection to graduate education. Thus, we are asking questions about the schools selection process and leadership competencies from basic medical education to senior doctor.

* 30. What support would be helpful for medical schools to integrate or assess leadership in the curriculum?

31. Does your medical school selection process select applicants against the below leadership domains? If yes, what competency/s are you selecting against?

Leads Self	
Engages Others	
Achieves Outcomes	
Drives Innovation	
Shapes Systems	
Not applicable	

* 32. Leads Self: At what education level do you believe the below leadership skills should be taught across the medical education continuum? (1. Day GE, Leggat SG. Leading and Managing Health Services: An Australian Perspective. 2015, Cambridge University Press. 2. Health LEADS Australia: The Australian Health Leadership Framework)

	Basic medical education	Junior doctor	Senior clinician
Ethical decision-making	☐	☐	☐
Self-management	☐	☐	☐
Emotional intelligence and self-awareness	☐	☐	☐
Exploring values	☐	☐	☐
Critical reflective practice	☐	☐	☐

* 33. Engages Others: At what education level do you believe the below leadership skills should be taught across the medical education continuum? (1. Day GE, Leggat SG. Leading and Managing Health Services: An Australian Perspective. 2015, Cambridge University Press. 2. Health LEADS Australia: The Australian Health Leadership Framework)

	Basic medical education	Junior doctor	Senior clinician
Communication	☐	☐	☐
Inter-professional teamwork	☐	☐	☐
Partnering with stakeholders	☐	☐	☐
Power in organisations	☐	☐	☐
Networking	☐	☐	☐

* 34. Achieves Outcomes: At what education level do you believe the below leadership skills should be taught across the medical education continuum? (1. Day GE, Leggat SG. Leading and Managing Health Services: An Australian Perspective. 2015, Cambridge University Press. 2. Health LEADS Australia: The Australian Health Leadership Framework)

	Basic medical education	Junior doctor	Senior clinician
Critical thinking and decision-making	☐	☐	☐
Managing staff	☐	☐	☐
Project management	☐	☐	☐
Financial management	☐	☐	☐
Negotiation	☐	☐	☐

* 35. Drives Innovation: At what education level do you believe the below leadership skills should be taught across the medical education continuum? (1. Day GE, Leggat SG. Leading and Managing Health Services: An Australian Perspective. 2015, Cambridge University Press. 2. Health LEADS Australia: The Australian Health Leadership Framework)

	Basic medical education	Junior doctor	Senior clinician
Creativity and visioning	☐	☐	☐
Evidence-based practice and use	☐	☐	☐
Successfully managing conflict	☐	☐	☐
Building positive workplace culture	☐	☐	☐
Leadership and management of change	☐	☐	☐
Quality and service improvement	☐	☐	☐

* 36. Shapes Systems: At what education level do you believe the below leadership skills should be taught across the medical continuum? (1. Day GE, Leggat SG. Leading and Managing Health Services: An Australian Perspective. 2015, Cambridge University Press. 2. Health LEADS Australia: The Australian Health Leadership Framework)

	Basic medical education	Junior doctor	Senior clinician
Workforce planning	☐	☐	☐
Strategic planning	☐	☐	☐
Health service planning	☐	☐	☐

* 37. Are there examples of good practice in your medical school of teaching or assessing leadership that you wish to share?

○ Yes

○ No

38. Please supply contact details below.

**Name**	
**University**	
**State/Province**	
**Email Address**	
**Phone Number**	

Medical Leadership Curricula, Assessment and Evaluation Survey

End

Thank you for your time and effort in completing this survey. At anytime you wish to be in contact, please email: simone.ross@jcu.edu.au

Please select the DONE button below to submit your completed survey.

Documents used to design this survey

1. Jefferies R, Ibrahim HN, Sheriff JH, et al. Leadership and Management in UK Medical School Curricula. Journal of Health Organization and Management. 2017;30(7)1081-1104

2. Day GE, Leggat SG. Leading and Managing Health Services: An Australasian Perspective. 2015. Cambridge University Press. ISBN: 9781316379684

3. Health LEADS Australia: the Australian Health Leadership Framework. https://www.aims.org.au/documents/item/352

4. Standards of Assessment and Accreditation of Primary Medical Program by the Australian Medical Council 2012. Page 4. http://www.amc.org.au/joomla-files/images/Accreditation/FINAL-Standards-and-Graduate-Outcome-Statements-20-December-2012.pdf
